# Supplementary material for: An Analysis of the Value of Information When Exploring Stochastic, Discrete Multi-Armed Bandits
Source: Entropy (Basel). 2018 Feb 28;20(3):155. doi: 10.3390/e20030155 (PMC7512671; doi:10.3390/e20030155)
Supplement: Supplementary file 1 [file entropy-20-00155-s001.zip › Entropy-237049-Supplementary Materials 2.pdf]

entropy

MDPI

Article

An Analysis of the Value of Information when Exploring Stochastic, Discrete Multi-Armed Bandits

Supplementary Materials 2

Isaac John Sledge [1,2]* and José Carlos Príncipe [1,2,3]*

[1] Department of Electrical and Computer Engineering, University of Florida, Gainesville, FL 32611, USA;
[2] Computational NeuroEngineering Laboratory (CNEL), University of Florida, Gainesville, FL 32611, USA;
[3] Department of Biomedical Engineering, University of Florida, Gainesville, FL 32611, USA
* Correspondence: isledge@ufl.edu (I.J.S.); principe@cnel.ufl.edu (J.C.P.)

Received: 12 October 2017; Accepted: 26 February 2018; Published: 28 February 2018

Supplementary Materials 2

In this supplementary, we further quantify the empirical capabilities of the value of information without hyperparameter tuning via cross entropy. The aims of our simulations are two-fold. First, we want to assess the average regret performance of our value-of-information-based search with state-of-the-art techniques. We utilize six additional algorithms for this purpose. Four of these algorithms, Bayesian UCB, KL-UCB, empirical KL-UCB, and CP-UCB, were chosen because they represent the best known extensions of UCB for the bandit problem that we consider. All four methods are known to achieve logarithmic regret with good constant factors. The fifth algorithm, DMED, was selected because it improves upon the asymptotic behavior of UCB. The last approach is the classic Thompson sampling, which, surprisingly, remains empirically competitive against large classes of bandit algorithms.

Secondly, we want to understand why certain cumulative average regrets were returned by these algorithms. Toward this end, we assess the average number of sub-optimal arm pulls.

S1. Simulation Preliminaries

The difficulty of the multi-armed bandit problem is fully characterized by two attributes: the distribution properties used to model the slot machines and the number of slot-machine arms.

For our simulations, the rewards for each of the slot-machine arms were sampled from Bernoulli distributions. The expected values for these distributions are uniformly sampled from the unit interval. We originally considered a range of additional reward scenarios, such as those that are bounded exponential, unbounded exponential, and bounded Poisson, but found that the relative performances between the different methods did not change much; we therefore only report the findings for the Bernoulli distributions.

Another aspect that changes the difficulty of the problem is the number of slot machines. We evaluate the algorithms for three, ten, and thirty slot machine arms. Three arms leads to fairly easy tasks, while ten arms furnishes marginally difficult ones. For Bernoulli rewards, over ten arms begins to provide tasks that are highly challenging, especially if the expectations of the rewards have a low variance. Originally, we considered greater numbers of slot machine arms. However, we found that their relative behaviors were mostly consistent with with the ten-armed case.

The value of information assumes that an initial policy is supplied. The results that we obtained in the paper indicate that regret is independent of the initial policy. Nevertheless, we specify that the initial policies have uniform probabilities, so as to not introduce bias that could lead to simpler problems. For all other approaches, we assume the same initialization process is followed, except in the instances where the algorithm explicitly states otherwise. For instance, some algorithms require that each of the arms be pulled once before beginning the exploration phase of the learning process.

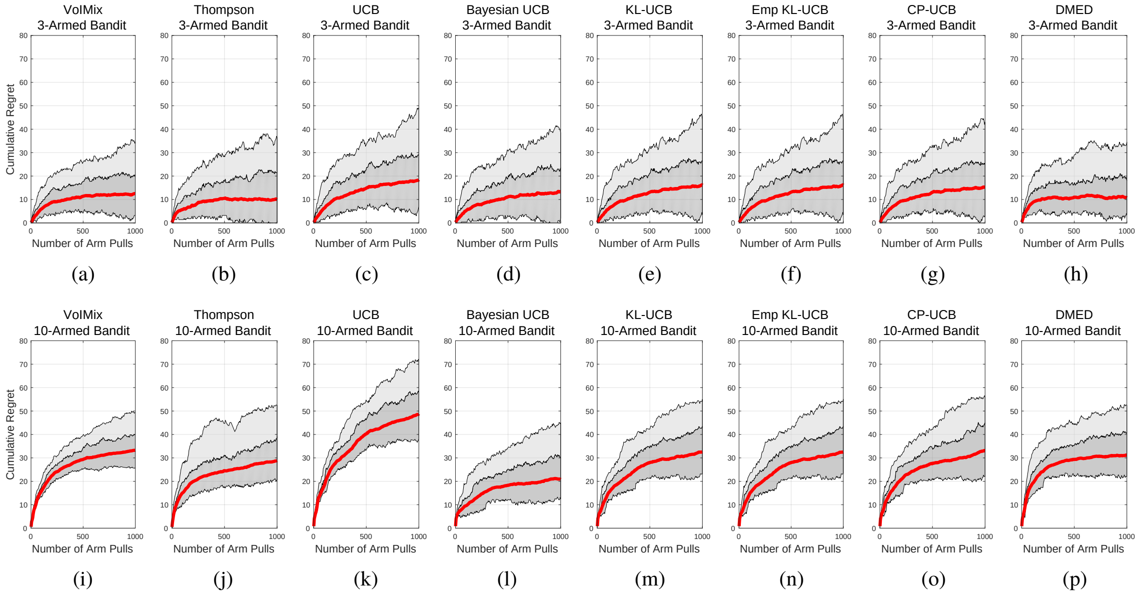

Figure A.1: Regret results for the Bernoulli-reward, multi-armed bandit problem. The plots in the first row, (a)–(h), correspond the application of different methodologies to the 3-armed bandit problem. The plots in the second row, (i)–(p), correspond the application of different methodologies to the 10-armed bandit problem. For each plot, the red curve corresponds to the cumulative regret across the arm pulls. The cumulative regret is averaged across the Monte Carlo simulations. The dark gray region corresponds to the first and third quartile of the average cumulative regret; this captures the lower and upper twenty-five percent of the regret spread across the simulations. The light gray region corresponds to the upper five-percent regret quantile. Low regret values correspond to good-performing methodologiies. Tight quantile bands indicate consistency of the reported cumulative regret.

S2. Simulation Results and Discussions

S2.1. Comparison Results

In what follows, we compare the performance of VoIMix against the different algorithms that we chose. Our simulation results, which comprise regret and the number of sub-optimal arm pulls, are provided in figures A.1 and A.2. For each of our simulations, we considered a thousand arm pulls, which captures the early search performance of these different algorithms and should be sufficient to reliably identify the optimal arm. We also considered averaging over a thousand Monte Carlo trials so as to represent the general trends in the exploration capabilities and filter out any overly abnormal runs.

Thompson Sampling. Thompson sampling [1] is one of the best-known stochastic algorithms for addressing the Bernoulli multi-armed bandit problem in a Bayesian fashion [2]. In this seminal work, Thompson proposed matching the probability of playing a particular arm with that arm's inherent probability of being the best, given the reward observed by sampling each distribution at least once and selecting the maximum sample.

The matching of arm-playing probabilities with the probability of being the best play can be formalized as follows. Given a set of parameters θ of the reward distribution, the probability of a given arm $a_k^i \in \mathcal{A}$ being the optimal one, at pull k, is expressed as follows: $\int \delta[\mathbb{E}[X_k^i | a_k^i, s_k, \theta] d\theta = \max_{a_k^j} \mathbb{E}][X_k^i | a_k^j, s_k, \theta]] p(\theta | s_{k-1}, a_{k-1}^i, X_{k-1}^i)$. Here, $s_{k-1}, a_{k-1}^i, X_{k-1}^i$ is an observation triple of the previous round, with $s_k \in \mathcal{S}$ being a context that the player receives. Rather than computing the integral using Monte Carlo techniques, Thompson and other researchers have shown that it suffices to simply sample the estimated, parametric pay-off distribution at each round and select the highest-sampled reward. That is, the repeated selection of the maximum of a single draw from each distribution produces an estimate of the optimal distribution.

The simplicity of Thompson sampling, along with its good empirical performance, has contributed to its adoption for a range of problems. Its wide-spread appeal, however, had been rather low until recent years, which

was was due to a lack of formal regret bounds. In the case where beta prior distributions are employed, Agrawal et al. [3] have shown that logarithmic regret can be obtained, albeit with somewhat poor constant factors. These results were improved by Kaufmann et al. [4] and Russo et al. [5], which indicate that Thompson sampling is, at the very least, comparable to correctly-tuned UCB-style methods.

Plots of the average cumulative regret for Thompson sampling are presented in figures A.1(b) and A.1(j) for the 3-armed and 10-armed bandit problems. Compared to UCB and tuned UCB, whose results were equivalent and provided in figures A.1(c) and A.1(k), Thompson sampling is far superior. On average, the regret is about a third to a half better, which is surprising, given the current best regret results. Thompson sampling often explores better than VoIMix, albeit not by much on average, which can be seen by contrasting figures A.1(b) and A.1(j) with A.1(a) and A.1(i). Curiously, the regret variance for Thompson sampling is higher than that for VoIMix, suggesting that sub-optimal arms may be pulled frequently in an attempt to construct the estimate of the optimal distribution. That is, Thompson sampling may be over exploring the action space in some instances, which is corroborated by the sub-optimal draw statistics reported in figures A.2(b) and A.2(j).

KL-UCB and CP-UCB. A promising state-of-the-art bandit algorithm, which has received marked attention in recent years, is Kullback-Leibler UCB (KL-UCB). KL-UCB was proposed by Lai and Robbins [6] and analyzed by Garivier and Cappé [7,8]. This approach improves upon the regret bounds of earlier UCB-style algorithms [9] by considering the divergence between the estimated distributions of each arm as a factor in the padding function p_i of UCB: $\arg\max_{a^i} \mu^i + p_i$, where $a^i \in \mathcal{A}$ is one of the i slot-machine arms.

For each episode of KL-UCB, arms $a^1, a^2, \ldots \in \mathcal{A}$ can be chosen by solving the following expression: $\arg\max_{a^i} n_i \mathrm{div}_{\mathrm{KL}}(\mu^i, \kappa) \leq \log(k) + c\log(\log(k))$, where κ is picked from the set of all possible reward distributions. Here, n_i is the number of times that arm $a^i \in \mathcal{A}$ has been played. Due to the incorporation of the Kullback-Leibler divergence $\mathrm{div}_{\mathrm{KL}}(\cdot,\cdot)$, this problem is strictly convex and increasing. The solution can hence be efficiently computed using a variety of existing techniques.

Various adaptations of KL-UCB have been considered for specific bandit problems. One of these is Clopper-Pearson UCB (CP-UCB), which is a specialization of KL-UCB to the case where Bernoulli rewards are used. CP-UCB differs from KL-UCB in the way that the upper-confidence bound on the performance of each arm is computed. That is, it chooses arms $a^1, a^2, \ldots \in \mathcal{A}$ by solving: $\arg\max_{a^i} u_{\mathrm{CP}}(r_i, n_i, k^{-1}\log(k)^{-c})$, where r_i is the cumulative reward for arm $a^i \in \mathcal{A}$ across the total number of pulls k and n_i is again the number of times that arm $a^i \in \mathcal{A}$ has been played. The function $u_{\mathrm{CP}}(\cdot,\cdot,\cdot)$ represents the Clopper-Pearson interval [10], which provides an exact method for calculating binomial confidence intervals.

Cumulative regret results for KL-UCB and an empirical variant of it are provided in figures A.1(e)–(f) and A.1(m)–(n). Outcomes for CP-UCB are given in figures A.1(g) and A.1(o). All of these algorithms typically produce worse regret than Thompson sampling. For the 3-armed bandit case, they noticeably lag behind VoIMix, which can be seen by comparing figure A.1(a) to figures A.1(e)–(g). For 10-armed bandits, the performance difference narrows, which is highlighted in figure A.1(i) and figures A.1(m)–(o). VoIMix is, on average, only marginally better than either KL-UCB or CP-UCB. Increasing the number of bandit arms beyond this range illustrated that KL-UCB and CP-UCB would often have average cumulative regrets that were par with VoIMix. In the event that the expected rewards were highly clustered, KL-UCB and CP-UCB tended to be better suited than VoIMix for finding the best-paying arm.

Bayesian UCB. Alongside KL-UCB, a Bayesian variant of UCB proposed by Kaufmann, Cappé and Garivier [11,12] represents the current state of the art in the exploration of stochastic, multi-armed bandits. For this approach, each arm is represented as an estimate of a distribution that resembles the upper confidence bounds in UCB. The arm with the best estimated score is chosen. The scoring process is modeled by a dynamic-in-time quantile of the posterior estimate.

More specifically, Bayesian UCB assumes that each arm has an associated prior distribution. This prior distribution is updated to an estimate of the posterior by computing quantiles of the expected distributions. The arm $a^i \in \mathcal{A}$ that maximizes the posterior quantile is chosen at iteration k: $\arg\max_{a^i} Q(1 - k^{-1}\log(K)^{-c}, \pi^i_{k-1})$. Here, π^i_{k-1} is the estimated posterior distribution of the arm $a^i_{k-1} \in \mathcal{A}$ chosen at iteration $k-1$. The function Q represents the quantile associated with the posterior distribution π^i_{k-1} at the previous iteration:

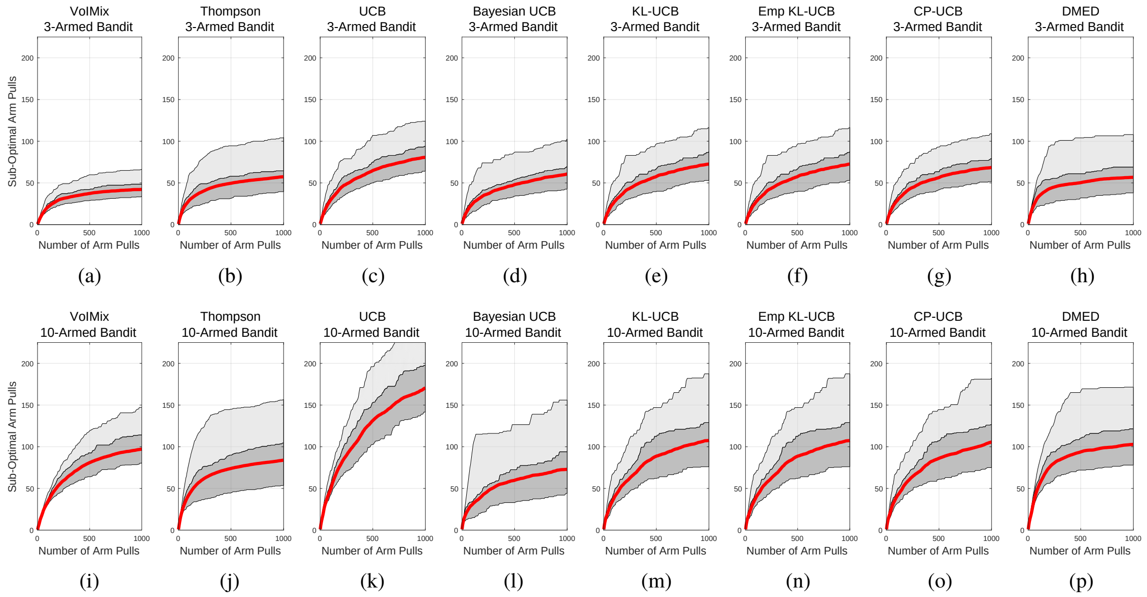

Figure A.2: Sup-optimal arm draw results for the Bernoulli-reward, multi-armed bandit problem. The plots in the first row, (a)–(h), correspond the application of different methodologies to the 3-armed bandit problem. The plots in the second row, (i)–(p), correspond the application of different methodologies to the 10-armed bandit problem. For each plot, the red curve corresponds to the average number of incorrect arm pulls made for the simulations. The dark gray region corresponds to the first and third quartile of the average number of incorrect arm pulls; this captures the lower and upper twenty-five percent of the sub-optimal draw spread across the simulations. The light gray region corresponds to the upper five-percent quantile. Low numbers of incorrect draws correspond to good-performing methodologiies. Tight quantile bands indicate algorithmic consistency in choosing the arms.

$p(X_{k-1}^i \leq Q(\cdot, \pi_{k-1}^i)) = 1 - k^{-1}\log(K)^{-c}$, with X_{k-1}^i being the reward at that round. The posterior distribution is then revised according to the Bayesian updating rule and then used as the prior for the next step.

Much like KL-UCB, Bayesian UCB has a cumulative regret that empirically outperforms the best of the original UCB algorithms by a substantial margin. In fact, in their theoretical analyses, Kaufmann et al. showed that Bayesian UCB achieves asymptotic optimality. Their approach also has the advantage of being distribution agnostic; it also does not rely on user-supplied information about the reward statistics.

Simulation results for the 3-armed and 10-armed bandit problems for Bayesian UCB are given in figures A.1(d) and A.1(l), respectively. Regardless of the difficulty of the problem, Bayesian UCB was either the best-performing or second best-performing algorithm out of those that we considered. On average, the regret was about a third better than either KL-UCB and its variants or VoIMix when the average rewards had a high variance. It additionally handled highly clustered expected rewards very well. In such cases, the average cumulative regret was between a half and a third that of the other UCB-style algorithms, which is a testament to the strong capabilities of Bayesian approaches when they utilize meaningful starting priors.

DMED. The final method that we consider, deterministic minimum empirical divergence (DMED) [13,14], improves upon the basic UCB approach by providing a means of achieving the theoretical asymptotic bound furnished by Lai and Robbins [6]. It does this in a manner similar to KL-UCB: by considering the divergence between the empirical distribution and the empirical mean of the best-paying arm that is currently found and using this divergence as a padding function. The arm that takes on the highest value of this padding function are chosen in each round.

DMED chooses arms $a^1, a^2, \ldots \in \mathcal{A}$ at each round by finding the arms that minimize: $n_i \mathrm{inf}_{\kappa_i} d_{\mathrm{KL}}(\overline{\mu}_k^*, \kappa_k^i) + \log(n_i) \leq \log(k)$. Here, κ_k^i is the empirical distribution found for the first k rounds from pulling arm $a^i \in \mathcal{A}$. $\overline{\mu}_k^* = \max_j \mu_k^j$ denotes the highest empirical mean after the first k arm pulls. As before, n_i denotes the number of times a particular arm has been played up to the current round. In essence, DMED attempts to minimize the posterior expectation of the regret. It does this by considering a term $n_i \mathrm{inf}_{\kappa_i} d_{\mathrm{KL}}(\overline{\mu}_k^*, \kappa_k^i)$ that corresponds to a

penalty for empirical distributions that are unlikely to occur from a distribution with expectation larger than $\overline{\mu}_k^*$. It also considers another term $\log(n_i)$ that penalizes arms which are pulled too many times; this second term acts as a main driver for exploration.

Results for DMED are presented in figures A.1(h), A.1(p), A.2(h) and A.2(p). As with KL-UCB and CP-UCB, DMED performed comparably to VoIMix. For highly simple problems, such as those with two arms, DMED actually had worse average regret. For problems with more than two arms, the expected cumulative regret was only slightly lower than that of VoIMix. An analysis of the sub-optimal arm draws, in figures A.2(h) and A.2(o) yielded a more interesting finding: there are simulations where DMED frequently tried poorly paying arms. In fact, the upper quantile bound suggests that non-optimal arms were tried about fifteen percent more often than for VoIMix, which is surprising. The only reason why DMED sometimes behaved better, on average, than VoIMix was because there were simulations where it consistently eschewed sub-optimal arms, thereby balancing out the effects of the poor runs.

S2.2. Comparison Discussions

These results indicate that the un-tuned version of VoIMix performs, in the short term, similarly to some of the state-of-the-art methodologies that we considered. Over a longer term it does too. There are some algorithms, however, that routinely outperformed VoIMix regardless of the number of arm pulls. These include Thompson sampling and Bayesian UCB.

Thompson sampling worked well, on average, because it carefully tracked the beliefs about the arms' possible pay-outs. That is, by sampling actions according to the posterior, the algorithm continues to consider and play all arms that could plausibly be optimal. While doing this, it shifts away from playing those arms those that are extremely unlikely to be optimal. Roughly speaking, the algorithm tries all promising actions while gradually discarding those that are believed to yield sub-par rewards. In some instances, however, it may not ignore poor arms quickly enough, which was captured by the quantile regions in figures A.2(b) and A.2(j).

VoIMix implements a similar behavior to Thompson sampling. That is, it experiences a period of pure exploration wherein all arms should, plausibly, be sampled more than once. This facilitates the estimation of the slot-machine expected pay-outs. After a certain number of pulls, the pure search phase gives way to the exploitation of the best-paying arm, as the exploration rate is iteratively decreased. It would appear, however, based upon the sub-optimal arm pull trends in figures A.2(a) and A.2(i), that the exploration phase may be too long compared to Thompson sampling. The number of sub-optimal arm pulls for Thompson sampling tapers off more quickly for higher number of arms, as indicated in figure A.2(j). Specifying a quicker annealing schedule may provide an adequate sampling of all of the arms and allow for the gambling strategy to focus on high-paying arms more quickly. This may help decrease the constant factor in our regret bound and improve the empirical performance of VoIMix. Alternatively, it may be necessary to account for the number of previous arm pulls, in VoIMix, when choosing which arm to play. This would ensure that all arms are played frequently enough to construct meaningful averages of the pay-outs. We plan to do the latter by revising the exponential terms in VoIMix, which yields a UCB-like algorithm.

Bayesian UCB provided excellent results for a few reasons. One of the most apparent is that Bayesian UCB can exploit the whole posterior distribution over the actions to determine which should be played. This becomes a major advantage when good priors are chosen for a given problem. That is, a great deal of insight can be conveyed, through the priors, about how to play the slot-machine arms if the form of the parametric reward distribution is known. The gambler can hence quickly settle on high-paying arms, as shown in figures A.2(d) and A.2(l). Although highest sub-optimal arm quantile for Bayesian UCB is comparable to other methods, its quartiles are often much better, which suggests that the beta priors that we chose were informative for the Bernoulli-based rewards. Another reason why Bayesian UCB performs well is that, as Kaufmann et al. have shown [11], it automatically constructs confidence intervals of the arm means that are adapted to the geometry of the problem. This helps the gambler to eschew otherwise good-performing arms, that are not optimal, much better and more quickly than other UCB-style approaches. This marked advantage is illustrated by comparing the results in figures A.2(e)–A.2(g) and A.2(m)–(o) to those in figures A.2(d) and A.2(l).

In his seminal work, Stratonovich had highlighted that the Bayesian-based value of information, when using Gaussian priors, has a algebraic solution for the two-state case [15]. In the future, we plan to consider a Bayesian version of the value of information to take advantage of these properties. Toward this end, we have already extended this result to both the multi-state and single-state case and shown that it is still possible to arrive at closed-form expressions. This formulation permits us to consider multi-armed bandit problems with Gaussian rewards from an entirely Bayesian context. It may also be possible to formulate closed-form expressions of this information-theoretic criterion for other parametric reward distributions. In doing so, we can definitively answer the following question: what is the benefit of performing a certain amount of exploration in this setting? It will also answer the question: how can we optimally perform this exploration when taking into account any relevant prior knowledge? This Bayesian framework may hence prove more fruitful than simply accounting for the number of times each arm has been played.

As shown in our simulations, VoIMix performs comparably to KL-UCB, CP-UCB, and DMED for this problem. This is despite the fact that such algorithms have a better constant factor in the regret expressions than our approach currently does. There are a few possible reasons why this occurred. Foremost, the regret expressions that have been derived for DMED and these UCB-style algorithms are asymptotic. For finite numbers of arm pulls, the instantaneous regret can be worse. Many thousands or tens of thousands more episodes beyond what we considered may be required before the empirical regret begins to align with the theoretical expectations. Another reason is that, as Kaufmann has noted [12], KL-UCB and its derivatives tend to over-explore the action space. This is because the exploration rate does not decrease in proportion with the number of times that an arm has been drawn. The same arm may hence be unnecessarily pulled multiple times, even if the expected reward estimate is good. The short-term regret may hence be artificially inflated. While VoIMix can suffer from the same issue, the results in figures A.2(a) and A.2(i) indicate that it was less slightly pronounced than in figures A.2(e)–A.2(h) and A.2(m)–A.2(p).

Somewhat surprisingly, CP-UCB failed to perform markedly better than either KL-UCB or empirical KL-UCB, let alone VoIMix, for the Bernoulli-reward bandit problem. This was despite CP-UCB being specifically formulated for such rewards. Similar findings were reported by Garivier and Cappé [8]. This could be due to the Clopper-Pearson interval estimation not providing coverage probabilities close to the nominal confidence level [16]. Better performance may be possible by substituting approximate schemes in place of the exact Clopper-Pearson method. Making this change would likely further sharpen the confidence intervals and make them more effective at discerning and ignoring poor-performing arms.

References

1. Thompson, W.R. On the likelihood that one unknown probability exceeds another in view of the evidence of two samples. *Biometrika* **1933**, *25*, 285–294.
2. Scott, S.L. A modern Bayesian look at the multi-armed bandit. *Applied Stochastic Models in Business and Industry* **2010**, *26*, 639–658.
3. Agarwal, S.; Goyal, N. Analysis of Thompson sampling for the multi-armed bandit problem. *Journal of Machine Learning Research* **2012**, *23*, 1–39.
4. Kaufmann, E.; Korda, N.; Munos, R. Thompson sampling: An asymptotically optimal finite-time analysis. Proceedings of the International Conference on Algorithmic Learning Theory; , 2012; pp. 199–213.
5. Russo, D.; Van Roy, B. Learning to optimize via posterior sampling. *Mathematics of Operations Research* **2014**, *39*, 1221–1243.
6. Lai, T.L.; Robbins, H. Asymptotically efficient adaptive allocation rules. *Advances in Applied Mathematics* **1985**, *6*, 4–22.
7. Garivier, A.; Cappé, O. The KL-UCB algorithm for bounded stochastic bandits and beyond. Proceedings of the Conference on Learning Theory (COLT); , 2011; pp. 359–376.
8. Cappé, R.; Garivier, A.; Maillard, O.A.; Munos, R.; Stoltz, G. Kullback-Leibler upper confidence bounds for optimal sequential allocation. *Annals of Statistics* **2013**, *41*, 1516–1541.
9. Auer, P.; Cesa-Bianchi, N.; Freund, Y.; Schapire, R.E. The nonstochastic multi-armed bandit problem. *SIAM Journal on Computing* **2002**, *32*, 48–77.

10. Clopper, C.J.; Pearson, E.S. The use fo confidence or fiducial limits illustrated in the case of the binomial. *Biometrika* **1934**, *26*, 404–413.
11. Kaufmann, E.; Cappé, O.; Garivier, A. On Bayesian upper confidence bounds for bandit problems. Proceedings of the International Conference on Artificial Intelligence and Statistics (AISTATS); , 2012; pp. 592–600.
12. Kaufmann, E. On Bayesian index policies for sequential resource allocation. *Annals of Statistics* **2016**. (under review).
13. Honda, J.; Takemura, A. An asymptotically optimal policy for finite support models in the multiarmed bandit problem. *Machine Learning* **2011**, *85*, 361–391.
14. Honda, J.; Takemura, A. Non-asymptotic analysis if a new bandit algorithm for semi-bounded rewards. *Journal of Machine Learning Research* **2015**, *16*, 3721–3756.
15. Stratonovich, R.L. *Information Theory*; Sovetskoe Radio: Moscow, Soviet Union, 1975.
16. Agresti, A.; Coull, B.A. Approximate is better than "exact" for interval estimation of binomial proportions. *American Statistician* **1998**, *52*, 119–126.

© 2018 by the authors. Licensee MDPI, Basel, Switzerland. This article is an open access article distributed under the terms and conditions of the Creative Commons Attribution (CC BY) license (http://creativecommons.org/licenses/by/4.0/).
