# Supplementary material for: An Analysis of the Value of Information When Exploring Stochastic, Discrete Multi-Armed Bandits
Source: Entropy (Basel). 2018 Feb 28;20(3):155. doi: 10.3390/e20030155 (PMC7512671; doi:10.3390/e20030155)
Supplement: Supplementary file 1 [file entropy-20-00155-s001.zip › Entropy-237049-Supplementary Materials 1.pdf]

entropy

MDPI

Article

An Analysis of the Value of Information when Exploring Stochastic, Discrete Multi-Armed Bandits

Supplementary Materials 1

Isaac John Sledge [1,2]* and José Carlos Príncipe [1,2,3]*

1 Department of Electrical and Computer Engineering, University of Florida, Gainesville, FL 32611, USA;
2 Computational NeuroEngineering Laboratory (CNEL), University of Florida, Gainesville, FL 32611, USA;
3 Department of Biomedical Engineering, University of Florida, Gainesville, FL 32611, USA
* Correspondence: isledge@ufl.edu (I.J.S.); principe@cnel.ufl.edu (J.C.P.)

Received: 12 October 2017; Accepted: 26 February 2018; Published: 28 February 2018

Supplementary Materials 1

In this supplementary, we provide supplemental simulation results for the tuned versions of VoIMix and AutoVoIMix. These results are given in Tables S1–S5.

© 2018 by the authors. Licensee MDPI, Basel, Switzerland. This article is an open access article distributed under the terms and conditions of the Creative Commons Attribution (CC BY) license (http://creativecommons.org/licenses/by/4.0/).

(a) Performance Difference, Increased Weighted-Random Exploration ($\tau_k = 0.1$ to $\tau_k = 1.0$)		
Rounds	Regret Change	Pay-out Change
0 to 50	$+47.47 \pm 7.99\%$	$-63.73 \pm 14.20\%$
51 to 150	$+48.79 \pm 4.96\%$	$-75.38 \pm 9.55\%$
151 to 250	$+53.78 \pm 4.12\%$	$-72.01 \pm 9.43\%$
(b) Performance Difference, Decreased Weighted-Random Exploration ($\tau_k = 0.1$ to $\tau_k = 0.01$)		
Rounds	Regret Change	Pay-out Change
0 to 50	$+13.04 \pm 1.77\%$	$+1.06 \pm 6.62\%$
51 to 150	$+26.10 \pm 1.82\%$	$-7.11 \pm 5.46\%$
151 to 250	$+31.63 \pm 1.58\%$	$-4.43 \pm 5.38\%$
(c) Performance Difference, Increased Uniform-Random Exploration ($\tau_k = 0.1$, $\epsilon_k = 0.16$ to $\epsilon_k = 0.33$)		
Rounds	Regret Change	Pay-out Change
0 to 50	$+19.56 \pm 3.86\%$	$-15.52 \pm 5.39\%$
51 to 150	$+29.02 \pm 3.34\%$	$-22.57 \pm 4.78\%$
151 to 250	$+34.13 \pm 3.25\%$	$-23.42 \pm 4.92\%$
(d) Performance Difference, Decreased Uniform-Random Exploration ($\tau_k = 0.1$, $\epsilon_k = 0.16$ to $\epsilon_k = 0.01$)		
Rounds	Regret Change	Pay-out Change
0 to 50	$+16.07 \pm 2.08\%$	$-6.67 \pm 4.22\%$
51 to 150	$+14.33 \pm 1.95\%$	$-2.15 \pm 4.03\%$
151 to 250	$+13.92 \pm 1.94\%$	$-1.64 \pm 3.92\%$

Table S1: VoIMix performance comparison when changing the amount of exploration. Results are averaged over the 3-, 10-, and 30-armed bandit cases. Positive regret percentages and negative pay-out percentages indicate that the change led to worse results.

(a) Performance Difference, Best Fixed-Parameter Case ($\tau_k = 0.1$, $\epsilon_k = 0.16$) versus Tuned VoIMix		
Rounds	Regret Change	Pay-out Change
0 to 5000	$-52.03 \pm 45.89\%$	$+137.31 \pm 79.18\%$
5001 to 15000	$-62.19 \pm 40.14\%$	$+144.21 \pm 66.40\%$
15001 to 25000	$-67.97 \pm 36.17\%$	$+151.10 \pm 49.72\%$
(b) Performance Difference, Best Fixed-Parameter Case ($\tau_k = 0.1$, $\epsilon_k = 0.16$) versus Tuned AutoVoIMix		
Rounds	Regret Change	Pay-out Change
0 to 5000	$+50.84 \pm 19.86\%$	$-7.89 \pm 22.12\%$
5001 to 15000	$+32.75 \pm 30.90\%$	$-4.73 \pm 18.60\%$
15001 to 25000	$-39.47 \pm 10.23\%$	$+33.57 \pm 10.56\%$

Table S2: Fixed-parameter VoIMix versus automatically-tuned-parameter VoIMix/AutoVoIMix performance comparison. Results are averaged over the 3-, 10-, and 30-armed bandit cases. Positive regret percentages and negative pay-out percentages indicate that the change led to worse results.

(a) Tuned VoIMix Performance Difference, Shorter Exploration Phase ($d \approx 0$)		
Rounds	Regret Change	Pay-out Change
0 to 5000	$-5.24 \pm 3.12\%$	$+8.63 \pm 4.27\%$
5001 to 15000	$+6.39 \pm 3.86\%$	$-10.04 \pm 6.05\%$
15001 to 25000	$+12.51 \pm 4.75\%$	$-18.95 \pm 7.84\%$
(b) Tuned VoIMix Performance Difference, Longer Exploration Phase ($d \approx \min_j \mu^* - \mu^j$)		
Rounds	Regret Change	Pay-out Change
0 to 5000	$+6.95 \pm 2.27\%$	$-4.32 \pm 2.95\%$
5001 to 15000	$+18.43 \pm 4.82\%$	$-21.91 \pm 4.93\%$
15001 to 25000	$+25.67 \pm 6.79\%$	$-32.07 \pm 6.45\%$

Table S3: VoIMix performance comparison when changing the exploration duration. Results are averaged over the 3-, 10-, and 30-armed bandit cases. Positive regret percentages and negative pay-out percentages indicate that the change led to worse results.

(a) Tuned AutoVoIMix Performance Difference, Longer Exploration Phase/Log Regret ($\theta \approx 0$)		
Rounds	Regret Change	Pay-out Change
0 to 5000	$+6.91 \pm 3.56\%$	$-9.13 \pm 5.12\%$
5001 to 15000	$+8.35 \pm 4.27\%$	$-11.42 \pm 6.87\%$
15001 to 25000	$+15.08 \pm 5.02\%$	$-23.20 \pm 6.41\%$
(b) Tuned AutoVoIMix Performance Difference, Shorter Exploration Phase/Log-Squared Regret ($\theta \approx 0.5$)		
Rounds	Regret Change	Pay-out Change
0 to 5000	$+5.73 \pm 2.56\%$	$-3.75 \pm 3.19\%$
5001 to 15000	$+19.65 \pm 4.92\%$	$-23.19 \pm 4.48\%$
15001 to 25000	$+24.38 \pm 6.08\%$	$-31.25 \pm 6.63\%$

Table S4: AutoVoIMix performance comparison when changing the exploration duration. Results are averaged over the 3-, 10-, and 30-armed bandit cases. Positive regret percentages and negative pay-out percentages indicate that the change led to worse results.

(a) VoIMix Performance Difference, Fixed Hyperparmeter Case versus Automatically Tuned		
Rounds	Regret Change	Pay-out Change
0 to 5000	$-19.43 \pm 5.26\%$	$+23.91 \pm 7.02\%$
5001 to 15000	$-25.15 \pm 5.81\%$	$+31.58 \pm 7.36\%$
15001 to 25000	$-29.09 \pm 6.09\%$	$+36.22 \pm 7.85\%$
(b) AutoVoIMix Performance Difference, Fixed Hyperparmeter Case versus Automatically Tuned		
Rounds	Regret Change	Pay-out Change
0 to 5000	$-10.82 \pm 5.12\%$	$+15.12 \pm 6.13\%$
5001 to 15000	$-14.38 \pm 5.50\%$	$+19.17 \pm 6.76\%$
15001 to 25000	$-18.21 \pm 6.14\%$	$+23.92 \pm 7.07\%$

Table S5: Fixed-hyperparameter versus automatically-tuned-hyperparameter performance comparison. Results are averaged over the 3-, 10-, and 30-armed bandit cases. Negative regret percentages and positive pay-out percentages indicate that the change led to better results.
